# Contralateral Facial Botulinum Toxin Injection in Cases with Acute Facial Paralysis May Improve the Functional Recovery: Where We Stand and the Future Direction

**DOI:** 10.29252/wjps.10.2.89

**Published:** 2021-05

**Authors:** Alireza Hamidian Jahromi, Petros Konofaos

**Affiliations:** 1 Department of Plastic and Reconstructive Surgery, Rush University Medical Center, Chicago, Illinois, USA;; 2 Department of Plastic Surgery, University of Tennessee Health Science Center, Memphis, Tennessee, USA.

**Keywords:** Facial paralysis, Botulinum toxin, Botox, Functional recovery, Outcome

## Abstract

Facial nerve (FN) impacts the function of the facial musculature by creating muscle tone at rest as well as the muscles’ voluntary and involuntary contractions. Temporary or permanent loss of FN function could be due to different etiologic factors. Acute facial paralysis (FP) could be significantly stressful for the patient and the family and apart from supportive measures management options are quite restricted. While botulinum toxin (BTX) injection in the FP setting has been used mostly to address the compensatory hyperkinesia in the non-paralyzed side of the face, there are evidence to suggest contralateral injection of the non-paralyzed face with BTX may improve/enhance the recovery time of the FP in cases where the FP has a reversible cause. While further studies are underway, using the current evidence as discussed could potentially justify the current usage of contralateral BTX injection and biofeedback exercises in the setting of the temporary FP specialty due to lack of effective alternative management options.

## INTRODUCTION

Facial nerve (FN) impacts the function of the facial musculature by creating muscle tone at rest as well as the muscles’ voluntary and involuntary contractions. By orchestrating complex synergistic and antagonistic forces, FN coordinates the function of multiple muscles for blinking reflex and corneal protection, oral sphincter function, understandable speech and emotional expressions including smile, laugh and anger. Temporary or permanent loss of FN function could be due to different etiologic factors including trauma, iatrogenic damage caused during operations, cerebral vascular dysfunctions, otitis media and viral infections, although idiopathic etiologies (paralysis of unknown origin) is considered as the most frequent type of facial paralysis (FP) especially in the temporary FP cases. 

FP could be significantly stressful for the patient and the family and apart from supportive measures including corneal protection, antiviral treatment in case of viral etiologies (although controversial due to conflicting data and uncertainty of benefits) and administration of the steroids in some special cases management options are quite restricted. Facial asymmetry is a hallmark of the FP cases. This asymmetry includes a decrease in wrinkling, less evident nasolabial fold and drooping of the eyebrow and the oral commissure on the affected side along with a compensatory hyperkinetic muscular reaction on the contralateral (normal) facial hemisphere^1^. This resultant facial deviation is more prominent when the patient actively attempts for a facial expression. De Maio and Bento used Botulinum toxin type A (BTX) injection (Dysport) mostly in the peri oral areas to address the compensatory hyperkinesia in 18 adult patients with chronic FP (more than one year of FP symptoms in their study) and followed the patients for evaluation of facial asymmetry for six months 1. 

In a quantitative analysis using digital caliper, they showed a significant reduction in hyperkinesis that lasted approximately six months as well as a 94% of patient satisfaction with results which started as early as two weeks after the disport injection^1^. The authors showed adverse events/complications including oral incontinence for liquids and solids, mostly due to abrupt blocking of the perioral muscles were transient and minimal in severity. While their study design was only focused on the improvement in hyperkinesia in the non-paralyzed side of the face, their patient selection (chronic non-reversible FP cases with at least one year of FP) did not allow them to observe changes in the recovery pattern of the FP. Bartoli et al.^2^ used contralateral BTX injections to improve facial symmetry and reduce contralateral hyperkinesis in six cases of moderate and severe FP (Brackmann score grade III to VI) with residual facial asymmetry and contralateral hyperkinesis after primary facial palsy surgery^2^. The authors showed improved facial symmetry, and aesthetic aspects along with a favorable result without widely common adverse events which lasted approximately 120 d in all of the patients^2^. While an improved functional facial recovery was reported, focus was mostly on managing the hyperkinesia in the non-paralyzed side and their small sample size (six patients) and lack of a control group did not allow them to evaluate the patients for a possible enhanced recovery of the FP side. 

In a prospective study in 17 patients with chronic (>1 year) unilateral FP used a combination of BTX and half-mirror biofeedback rehabilitation for about two years. The results showed long-lasting improvement in facial synkinesis, aesthetics and symmetry in the patients^3^. Interestingly in a prospective study, 42 patients with partial recovery were evaluated from FP and injected both sides of the patients’ faces (paralyzed and non-paralyzed sides) for treatment of post-paralytic facial synkinesis in an attempt to “produce a new balance”. Significant improvement of the synkinesis, food intake, social interaction and quality of life and enhancement of facial symmetry and personal appearance were reported during the follow-up^4^. Again, the study design did not include a comparison arm to see whether contralateral injection of the non-paralyzed face with BTX made any impact on the recovery time of the FP or if concurrent injection of the paralyzed side with BTX made any additional/enhanced recovery impact. 

Kim explored the impact of the BTX injection in improving the asymmetry in FP patients with a focus on the patients with acute FP (nine cases of Bell’s palsy with age range of 22-82 yr). While during the recovery period patients with acute FP are advised to only wait for the spontaneous recovery with no effective intervention being present, this may not be true^5^. Kim showed a significant relief of asymmetry in eight of the observed nine cases within four weeks from injection. The improved symmetry was confirmed by both physician and the patient following BTX injection after long-standing FP^6^. An increase was shown in patient’s public confidence and psychologic aspects as well as improvement in articulation, eating, drinking, and a reduction in their cosmetic embarrassment. 

The hallmark study included 65 adult rats and evaluated the impact of temporary chemical denervation using BTX on the contralateral non-paralyzed hemiface and showed not only an improved functional recovery of the whisker pads but also an increase in regeneration of the lesioned buccal branch of the facial nerve^7^. Mis-directional axonal sprouting and poly-innervation of the endplate nerve terminals are parts of the facial nerve recovery, even in the optimal nerve reconstruction and nerve recovery. The authors distributed the cases in four interventional arms including FN anastomosis, FN anastomosis and ipsilateral BTX injection, FN anastomosis and contralateral injection, BTX injection with no FN surgery^7^. Using the retrograde fluorescence tracing, Guntinas-Lichius quantified the accuracy of reinnervation and measured the functional recovery by biometrical image analysis of whisking behavior after surgery. While ipsilateral or contralateral BTX injection both increased the correct reinnervation from 27% to 61%, enhanced correct reinnervation accompanying the improved functional recovery (whisking) was seen only with contralateral but not ipsilateral injection of BTX^7^. Guntinas-Lichius and colleagues concluded that the morphological as well as functional regeneration of the FN is seen on the paralyzed side when the clinician injects the non-paralyzed side of the face in cases with FP or following FN repair^7^. 

Although this observation seems difficult to fully explain, Guntinas-Lichius proposed some possible explanation while admitting their study design failed to evaluate the role of the supraspinal centers controlling locomotion of the whiskers in rats (FN function)^7^. While ipsilateral injection of the BTX improved the proper motoneuronal reinnervation, it failed to improve the FN function and recovery compared with contralateral BTX injection^7^. An increase in activation of the contralateral hemisphere (motor cortex of the pertinent paralyzed FN) secondary to temporary under activation of the ipsilateral motor cortex (motor cortex of the pertinent non-paralyzed FN) following BTX injection may be a potential explanation for better functional results. This increased activation in their opinion could simply represent the absence of transcallosal inhibition from the contralateral hemisphere. A compensatory activity in the dorsal premotor cortex supporting the functional motor reorganization and regeneration could be another possibility^7^. An important positive point about BTX administration in the setting of the FP is continuation of the sensory inputs, especially as the enforcement impact of the sensory input on the FN regeneration has been documented before^7^. Even in the setting of the peripheral nerves (i.e. forearm, hand and leg muscles), sensory input improves regeneration of the peripheral nerves and modulates excitability of both ipsilateral and contralateral motor cortices simultaneously^8^^-^^10^. A rodent experimental model was used and studied the impact of contralateral leg (gastrocnemius muscle) BTX injection following a tibial nerve (peripheral nerve in the lower extremity) lesion and repair in rats. The authors showed similar type of enhanced functional recovery in rats following BTX injection to the contralateral leg following the section and repair of the tibial nerve^9^. Lima et al. attributed their observation to the “neuroplasticity phenomenon” and an increase in the cortical activity of bilateral hemispheres of the brain following a peripheral nerve conduction blockage ^9^. The fact that ipsilateral injection of the BTX in the Guntinas-Lichius experiment^7^ improved the proper motoneuronal reinnervation, but failed to improve the FN function and recovery compared with contralateral BTX injection brings us to the point that the “neuroplasticity phenomenon” is mostly beneficial and important when the BTX is injected on the contralateral side of the limb or face and allows the cortex pertinent to the paralyzed side becoming more active. 

## CONCLUSION

We currently lack randomized controlled trial in humans evaluating the impact of BTX injection during facial nerve regeneration both on terminal sprouting and on the ipsilateral and contralateral sensorimotor cortex with good documentation of the brain cortical function. This provisional information along with evaluation of the additional impact of the “Half-mirror biofeedback” exercise with contralateral BTX injection in the setting of the acute FP could potentially become the standard of care for management of temporary FP management. Extended safety profile of the BTX injection along with lack of imaginable side effects of the “Half-mirror biofeedback” exercise in the setting of FP makes the transition from the experimental stage to the widespread clinical implementation of these managements very fast and smooth. While further studies are underway, using the current evidence as discussed could potentially justify the current usage of contralateral BTX injection and biofeedback exercises in the setting of the temporary FP specialty due to lack of effective alternative management options. 
